# Cost-utility analysis of loxenatide versus DPP-4 inhibitors in Chinese patients with type 2 diabetes at high cardiovascular risk

**DOI:** 10.3389/fpubh.2026.1929706

**Published:** 2026-08-28

**Authors:** Longbing Lai, Fengning Chuan, Qiuhong Huang, Na Xiong, Chuan Peng, Ruobei Zhao, Jianjuan Sun, Qiang Liu, Jiangang Lu

**Affiliations:** 1 Department of Endocrinology, Chongqing Municipal Key Clinical Specialty, Chongqing University FuLing Hospital, Chongqing, China; 2 School of Business Administration, Shenyang Pharmaceutical University, Shenyang, Liaoning, China

**Keywords:** cost-utility analysis, FIGHTING-2 trial, high cardiovascular risk, Markov model, sensitivity analysis, T2DM

## Abstract

**Background:**

In China, many patients with type 2 diabetes mellitus (T2DM) face high cardiovascular risk and a heavy disease burden. Loxenatide, a once-weekly GLP-1 receptor agonist developed in China, is now listed for reimbursement, but evidence on its long-term cost-effectiveness is limited. The FIGHTING-2 study, presented at the 2026 ECIM, provided the first direct comparison of cardiovascular outcomes between loxenatide and a DPP-4 inhibitor (sitagliptin) in Chinese T2DM patients at high cardiovascular risk and supplied core efficacy parameters for this study.

**Objective:**

Assess the long-term cost-utility of loxenatide (0.2 mg once weekly) versus sitagliptin (100 mg once daily) in high cardiovascular risk type 2 diabetes patients in China inadequately controlled with metformin, from the healthcare system perspective.

**Methods:**

A five-state Markov model (event-free, myocardial infarction, stroke, heart failure, and death) was developed to simulate the natural disease progression of patients with type 2 diabetes at high cardiovascular risk. The model used a 1-year cycle length over a 40-year time horizon. Transition probabilities were estimated using the UKPDS 82 risk equations, and clinical efficacy inputs were obtained from the FIGHTING-2 study. Cost and utility parameters were derived from Chinese local literature and the National Reimbursement Drug List. The willingness-to-pay threshold was set at three times China’s per capita GDP in 2025 (299,100 CNY per QALY), and both costs and health outcomes were discounted at an annual rate of 5%. The incremental cost-effectiveness ratio (ICER) was calculated in the base-case analysis. One-way and probabilistic sensitivity analyses (1,000 Monte Carlo simulations) were conducted to assess the robustness of the results.

**Results:**

In the base-case analysis, loxenatide showed an ICER of 82058.77 CNY per QALY compared with sitagliptin, which is well below the willingness-to-pay threshold (299,100 CNY per QALY). One-way sensitivity analysis showed that the ICER was most affected by the hazard ratio for heart failure and by loxenatide cost, but loxenatide remained cost-effective across all scenarios. Probabilistic sensitivity analysis indicated loxenatide was cost-effective in 100% of simulations and consistently generated additional QALYs, demonstrating robust model results. The scenario analysis also confirmed the robustness of the model results.

**Conclusion:**

Loxenatide has a clear cost-utility advantage over sitagliptin in the treatment of Chinese patients with type 2 diabetes at high cardiovascular risk. These findings provide high-quality evidence to support clinical standardization, rational drug use, reimbursement policy decisions, and optimal allocation of healthcare resources for this patient population in China.

## 1 Introduction

Type 2 diabetes mellitus (T2DM) is a major global public health challenge. According to the International Diabetes Federation, there were 537 million adults with diabetes worldwide in 2021, with China having the largest diabetic population (approximately 141 million) (1). Cardiovascular disease (CVD) is the leading cause of death in T2DM patients, accounting for over half of diabetes-related deaths (2). Heart failure and stroke also contribute significantly to poor prognosis and increased medical costs (3). A large proportion of Chinese T2DM patients are at high cardiovascular risk. Thus, achieving glycemic control while managing multiple risk factors and reducing long-term complications has become a core clinical priority.

Although metformin is recommended as first-line therapy, many patients do not achieve target HbA1c (<7.0%) on metformin alone and require second-line treatment (4). Traditional second-line agents such as dipeptidyl peptidase-4 inhibitors (DPP-4i, e.g., sitagliptin) are safe and convenient but offer limited benefits in weight control, blood pressure improvement, and cardiorenal protection (5). In contrast, glucagon-like peptide-1 receptor agonists (GLP-1RAs) provide potent glucose-lowering, weight-reduction, blood-pressure-lowering, lipid-lowering, and cardiorenal-protective effects, leading to their preferential recommendation for T2DM patients with atherosclerotic CVD, heart failure, or obesity (6, 7).

Loxenatide (polyethylene glycol loxenatide, PEG-Loxe) is a once-weekly long-acting GLP-1RA developed in China, approved by the National Medical Products Administration, and included in the National Reimbursement Drug List. Previous studies have confirmed its efficacy in reducing HbA1c, body weight, blood pressure, and lipids, with convenient once-weekly dosing that may improve adherence (8, 9). However, its cardiovascular effects were previously unknown. At the 2026 European Congress of Internal Medicine (ECIM), the FIGHTING-2 study reported the first head-to-head real-world comparison of loxenatide versus a DPP-4 inhibitor (sitagliptin) in Chinese patients with T2DM and high cardiovascular risk. This multicenter, retrospective real-world cohort study used data from the Tianjin Health Data Platform covering 82 hospitals. Propensity score matching (PSM) was applied at a 1:1 ratio to adjust for age, sex, diabetes duration, CVD history, dyslipidemia, hypertension, kidney disease, and concomitant use of SGLT2 inhibitors, antihypertensive agents, and lipid-lowering drugs, resulting in 1,972 patients per group from the total screened population (10). The study found that the incidence of three-point major adverse cardiovascular events (3P-MACE: all-cause death, non-fatal myocardial infarction, non-fatal stroke) was 1.9% in the loxenatide group versus 4.5% in the DPP-4i group, representing a 42% risk reduction (HR = 0.58, 95% CI: 0.39–0.86, *p* = 0.007). Loxenatide also reduced non-fatal stroke by 48% (HR = 0.52, 95% CI: 0.33–0.83, *p* = 0.006), heart failure hospitalization by 68% (HR = 0.32, 95% CI: 0.15–0.68, *p* = 0.003), and showed a trend toward lower all-cause mortality (HR = 0.29, 95% CI: 0.07–1.27, *p* = 0.099). No significant difference was observed in non-fatal myocardial infarction (HR = 1.00, 95% CI: 0.49–2.41, *p* = 0.834) (10). This is the first real-world study demonstrating that loxenatide significantly reduces 3P-MACE, non-fatal stroke, and heart failure hospitalization in Chinese T2DM patients at high cardiovascular risk, providing high-level evidence for its cardiovascular protection.

Despite these encouraging clinical results, the long-term cost-effectiveness of loxenatide versus sitagliptin remains unknown. Healthcare resources in China are limited, and clinical efficacy alone cannot guide reimbursement and prescribing decisions; local pharmacoeconomic evidence is needed. To date, no cost-utility analysis comparing loxenatide and sitagliptin has been conducted in the Chinese population. Therefore, from the Chinese healthcare system perspective, we developed a five-state Markov model to simulate the lifelong disease course and evaluate the cost-effectiveness of loxenatide (0.2 mg once weekly) versus sitagliptin (100 mg once daily) in T2DM patients at high cardiovascular risk, aiming to provide high-quality local evidence for rational drug use, reimbursement decisions, and healthcare resource allocation.2 Materials and methods.

### 1.1 Target population

The target population of this study was patients with type 2 diabetes at high cardiovascular risk. Inclusion criteria were mainly based on the FLYING trial (a multicenter ambispective cohort study of loxenatide in China) (11) and the target population definition of the FIGHTING-2 study (12), as follows: ① age ≥18 years; ② confirmed diagnosis of type 2 diabetes; ③ presence of one or more cardiovascular diseases or risk factors (including coronary heart disease, stroke/transient ischemic attack, peripheral artery disease, hypertension, dyslipidemia, obesity (BMI ≥ 28 kg/m^2^), smoking, etc.); ④ inadequate glycemic control (HbA1c 7.0–10.5%) despite stable metformin therapy (≥1,500 mg/day or maximum tolerated dose ≥1,000 mg/day). Exclusion criteria included type 1 diabetes, gestational diabetes, severe liver or kidney dysfunction, malignancy, known allergy to study drugs, and other conditions explicitly excluded in the FLYING trial (11). These inclusion criteria ensured that enrolled patients had clearly defined high cardiovascular risk, consistent with the population on which the hazard ratios from the FIGHTING-2 study were based, thereby supporting the extrapolation of those hazard ratios to the present model.

### 1.2 Model structure

**Figure 1:**
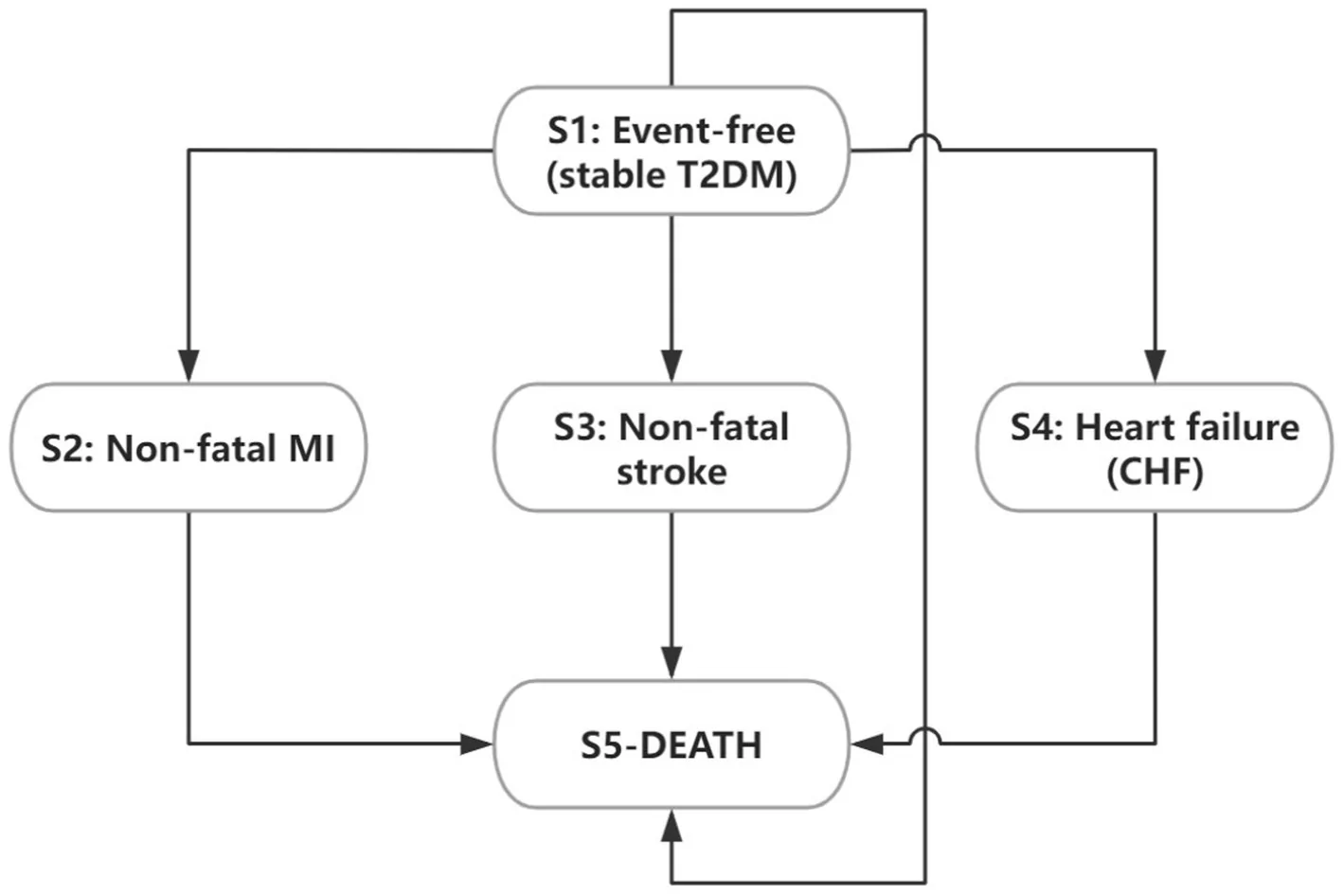
Markov model diagram.

A five-state Markov model was constructed, consisting of: S1 (event-free, stable type 2 diabetes), S2 (first non-fatal myocardial infarction, MI), S3 (first non-fatal stroke), S4 (first hospitalization for heart failure, CHF), and S5 (death, an absorbing state). Patients started in S1, and state transitions followed the natural disease course: S1 could transition to S2, S3, S4, or S5; S2, S3, and S4 could also transition directly to S5; no transitions between non-absorbing states were allowed (i.e., only the first cardiovascular event was considered) (Figure 1).

This five-state structure reflects the major clinical pathways of patients with type 2 diabetes at high cardiovascular risk. The event-free state (S1) corresponds to the stable period preceding any cardiovascular event. The first non-fatal MI (S2), first non-fatal stroke (S3), and first hospitalization for heart failure (S4) are the core cardiovascular outcomes of interest, directly corresponding to the hazard ratios reported in the FIGHTING-2 trial. The death state (S5) was set as an absorbing state. Recurrent events or concurrent multiple events were not included because the treatment strategy and risk profile change fundamentally after the first event. The main adverse reactions of loxenatide and sitagliptin (gastrointestinal symptoms) are mostly mild to moderate, self-limiting, and similar in incidence between the two groups. Therefore, consistent with pharmacoeconomic modeling conventions, adverse events were not separately modeled (13).

The model cycle length was set to 1 year, with a 40-year time horizon. A half-cycle correction was applied to better approximate the continuous occurrence of events within cycles. The analysis was conducted from the perspective of the Chinese healthcare system, focusing only on direct medical costs. Following the recommendations of the China Guidelines for Pharmacoeconomic Evaluations (2020 Edition) (14), the willingness-to-pay (WTP) threshold was set at three times the per capita GDP of China in 2025 (99,700 CNY), i.e., 299,100 CNY per QALY, and both costs and health outcomes were discounted at an annual rate of 5% (15). To eliminate random error and ensure the stability of Monte Carlo simulations, a cohort size of 10,000 patients was used, following the approach of Gu et al. (16).

### 1.3 Markov model parameters

#### 1.3.1 Patient baseline characteristics

The initial cohort consisted of 10,000 Chinese adults with type 2 diabetes at high cardiovascular risk. Baseline characteristics and their data sources are presented in Table 1.

**Table 1:** Baseline characteristics of the patient cohort.

Parameter	Symbol	Value	Source
Starting age (years)	Age_start	53.1	(25)
Starting diabetes duration (years)	Dur_start	6.38	(25)
Proportion of female	prop_female	0.43	(25)
Proportion of smokers	prop_smoke	0.22	(26)
Baseline HbA1c (%)	HbA1c_base	8.1	(25)
Baseline systolic blood pressure (mmHg)	SBP_base	128.8	(25)
Baseline LDL-C (mmol/L)	LDL_base	2.89	(25)
Baseline BMI (kg/m^2^)	BMI_base	27.5	(25)

#### 1.3.2 Treatment effects and hazard ratios

The control group received sitagliptin (100 mg once daily). The experimental group received loxenatide (0.2 mg once weekly subcutaneously). The FIGHTING-2 study has only been published as an abstract. It did not report specific differences between loxenatide and sitagliptin in HbA1c, systolic blood pressure, or body weight. We therefore directly used the hazard ratios (HRs) reported in that study to adjust for the risks of cardiovascular events and death (12). HR for myocardial infarction = 1.00 (95% CI was not reported in the original study; in the sensitivity analysis, this parameter was tested over a wide range referencing values from similar studies.), HR for stroke = 0.52 (95% CI 0.40–0.98), HR for heart failure hospitalization = 0.32, and HR for all-cause death = 0.29 (95% CI 0.27–1.16). We multiplied these HRs by the annual transition probabilities derived from the UKPDS 82 equations for the control group. This gave the annual transition probabilities for the experimental group (Table 2).

**Table 2:** Hazard ratios for treatment effects.

Group	MI HR	Stroke HR	HF HR	Death HR	Source
Loxenatide vs. DPP-4i	1.00	0.52	0.32	0.29	(12)
DPP-4i (control)	1.00	1.00	1.00	1.00	Reference

#### 1.3.3 Transition probabilities

##### 1.3.3.1 Overview of the UKPDS 82

The UKPDS 82 (United Kingdom Prospective Diabetes Study 82) risk equations were developed based on 30-year follow-up data from the UK Prospective Diabetes Study. These equations estimate the annual probability of first occurrence of myocardial infarction, stroke, heart failure, and death without complications in patients with type 2 diabetes. The equations incorporate covariates such as age, diabetes duration, sex, HbA1c, systolic blood pressure, low-density lipoprotein cholesterol (LDL-C), body mass index (BMI), and smoking status to construct linear predictors. Survival functions for different events are fitted using exponential, Weibull, or Gompertz distributions (17).

In the UKPDS 82 equations, the cumulative hazard functions for each event are parameterized as described. Following the approach of a published Chinese pharmacoeconomic study (16), we first calculated cumulative hazards using the UKPDS 82 equations and then converted them to annual probabilities. These annual probabilities were used as transition probabilities for the control group (sitagliptin) in the Markov model.

##### 1.3.3.2 Calculation of cumulative hazard and annual probability

In the survival analysis framework, the cumulative hazard function H(t) represents the cumulative hazard intensity from baseline (t = 0) to time t, while the annual event probability P(t) denotes the probability of a first event occurring in the interval [t,t+1) . The relationship between the two is as follows:


P(t)=1−exp{−[H(t+1)−H(t)]} (1)

In this study, the cycle length was set to 1 year, and P(t) for each event was calculated for every simulation year. The cumulative hazard functions for each event and the resulting annual probability formulas are presented below.

###### 1.3.3.2.1 Probability of myocardial infarction

According to the UKPDS 82 equations, the risk of MI differs between males and females; therefore, the probabilities were calculated separately and then weighted by sex. For males, MI follows an exponential distribution (i.e., a constant instantaneous hazard rate), and the cumulative hazard function is calculated as follows:


$$H_{\mathrm{MI},\text{male}}(t)=e^{\lambda_{\mathrm{MI},\text{male}}+\sum \beta_{i}x_{i}}\cdot t$$

Where $\lambda_{\mathrm{MI},\text{male}}=-8.791$ , and $\sum \beta_{i}x_{i}$ is the sum of products of the regression coefficients and the covariates (including age, HbA1c, LDL-C, systolic blood pressure, and smoking status). From Equation 1, the annual probability is given by:


$$P_{\mathrm{MI},\text{male}}=1-\exp(-e^{\lambda_{\mathrm{MI},\text{male}}+\sum \beta_{i}x_{i}})$$

For female myocardial infarction (Weibull distribution), the cumulative hazard function is:


$$H_{\mathrm{MI},\text{female}}(t)=e^{\lambda_{\mathrm{MI},\text{female}}+\sum \beta_{i}x_{i}}\cdot t^{\rho_{\mathrm{MI},\text{female}}}$$

Where $\lambda_{\mathrm{MI},\text{female}}=-8.708$ , $\rho_{\mathrm{MI},\text{female}}=1.376$ . The annual probability is then:


$$P_{\mathrm{MI},\text{female}}(t)=1-\exp\{-e^{\lambda_{\mathrm{MI},\text{female}}+\sum \beta_{i}x_{i}}[{\left(t+1\right)}^{\rho_{\mathrm{MI},\text{female}}}-t^{\rho_{\mathrm{MI},\text{female}}}]\}$$

Given that the proportion of females in the target population baseline is 0.43 (Table 1), the proportion of males is 0.57. Therefore, the annual probability of myocardial infarction for the total population is given by:


$$P_{\mathrm{MI},\text{total}}(t)=0.57\cdot P_{\mathrm{MI},\text{male}}+0.43\cdot P_{\mathrm{MI},\text{female}}(t)$$

This probability is used as the annual transition probability from the event-free state (S1) to the myocardial infarction state (S2) in the Markov model.

###### 1.3.3.2.2 Stroke

According to the UKPDS 82 equations, the cumulative hazard function for stroke follows a Weibull distribution, and is expressed as:


$$H_{\text{Stroke}}(t)=e^{\lambda_{\text{Stroke}}+\sum \beta_{i}x_{i}}\cdot t^{\rho_{\text{Stroke}}}$$

Where $\lambda_{\text{Stroke}}=-13.053$ and $\rho_{\text{Stroke}}=1.466$ , The annual probability is:


$$P_{\text{Stroke}}(t)=1-\exp\{-e^{\lambda_{\text{Stroke}}+\sum \beta_{i}x_{i}}[{\left(t+1\right)}^{\rho_{\text{Stroke}}}-t^{\rho_{\text{Stroke}}}]\}$$

###### 1.3.3.2.3 Heart failure

According to the UKPDS 82 equations, the cumulative hazard function for heart failure follows a Weibull distribution, expressed as:


$$H_{\mathrm{CHF}}(t)=e^{\lambda_{\mathrm{CHF}}+\sum \beta_{i}x_{i}}\cdot t^{\rho_{\mathrm{CHF}}}$$

Where $\lambda_{\mathrm{CHF}}=-12.332$ and $\rho_{\mathrm{CHF}}=1.514$ , The annual probability is:


$$P_{\mathrm{CHF}}(t)=1-\exp\{-e^{\lambda_{\mathrm{CHF}}+\sum \beta_{i}x_{i}}[{\left(t+1\right)}^{\rho_{\mathrm{CHF}}}-t^{\rho_{\mathrm{CHF}}}]\}$$

###### 1.3.3.2.4 Death without complications

According to the UKPDS 82 equations, the cumulative hazard function for death without complications follows a Gompertz distribution, expressed as:


$$H_{\text{Death}}(t)=\frac{1}{\varphi }e^{\lambda_{\text{Death}}+\sum \beta_{i}x_{i}}\cdot (e^{\mathrm{\varphi t}}-1)$$

where $\lambda_{\text{Death}}=-10.908$ and φ=0.098 , and t represents current age. The annual probability is:


$$P_{\text{Death}}(t)=1-\exp\{-\frac{1}{\varphi }e^{\lambda_{\text{Death}}+\sum \beta_{i}x_{i}}\cdot [e^{\varphi (t+1)}-e^{\mathrm{\varphi t}}]\}$$ (2)

In the above calculations, patients’ age and diabetes duration increase by 1 year each cycle, while other risk factors (HbA1c, systolic blood pressure, LDL-C, BMI, and smoking status) are assumed to remain at their baseline values.

##### 1.3.3.3 Death risk for patients with a prior event

Since the UKPDS 82 equations are only applicable to event-free individuals and cannot directly estimate the subsequent annual mortality risk for patients who have experienced a myocardial infarction, stroke, or heart failure, we adjusted the post-event mortality probability using hazard ratios (HRs) derived from relevant literature and clinical experience.

After myocardial infarction: Based on the China Acute Myocardial Infarction Registry (CAMIRegistry) (18), the 2-year all-cause mortality HR for diabetic patients with acute myocardial infarction and a high stress hyperglycemia ratio was 1.73 (95% CI:1.39–2.15). Accordingly, we set the post-MI HR to 1.73.

After stroke: Based on a quality improvement study from the Chinese Stroke Center Alliance (CSCA) (19), the adjusted odds ratio for in-hospital all-cause mortality in diabetic/suspected diabetic patients with acute ischemic stroke was 1.30 (95% CI:1.23–1.38). Given that odds ratios can approximate HRs when event rates are high, we set the post-stroke HR to 1.30.

After heart failure: Due to the lack of high-quality published literature directly reporting long-term mortality HRs for Chinese T2DM patients after heart failure, we conservatively set the post-HF HR to 1.73, based on the findings of the Kailuan cohort studies (20, 21) and the clinical severity of heart failure. In sensitivity analyses, this value was varied over a wide range (1.30–2.50).

The annual post-event mortality probability was calculated as:


$$P_{\text{post}-\text{event}}=P_{\text{Death}}(t)\times HR_{\text{event}}$$

Where $P_{\text{Death}}(t)$ is the annual mortality probability for event-free individuals in the same cycle (Equation 2). The above transition probability calculations and mortality risk adjustments were applied to both the control and experimental groups. The annual event probabilities for the experimental group were further multiplied by the corresponding treatment effect HRs (Table 2) to account for the additional risk reduction of loxenatide compared with sitagliptin. The resulting transition probabilities for each health state are shown in Table 3.

**Table 3:** Annual transition probability table for each state.

Year	MI (Sitagliptin)	Stroke (Sitagliptin)	CHF (Sitagliptin)	Death (Sitagliptin)	MI (Loxenatide)	Stroke (Loxenatide)	CHF (Loxenatide)	Death (Loxenatide)
1	0.012	0.0045	0.005	0.0034	0.012	0.0024	0.0016	0.001
5	0.0158	0.0073	0.0083	0.0051	0.0158	0.0038	0.0027	0.0015
10	0.0214	0.0121	0.0142	0.0083	0.0214	0.0063	0.0045	0.0024
15	0.0283	0.019	0.0229	0.0135	0.0283	0.0099	0.0073	0.0039
20	0.0367	0.0291	0.0356	0.022	0.0367	0.0151	0.0114	0.0064
25	0.0471	0.0436	0.0543	0.0356	0.0471	0.0227	0.0174	0.0103
30	0.06	0.0644	0.0813	0.0574	0.06	0.0335	0.026	0.0167
35	0.0759	0.0936	0.1196	0.092	0.0759	0.0487	0.0383	0.0267
40	0.0952	0.1344	0.1729	0.1458	0.0952	0.0699	0.0553	0.0423

#### 1.3.4 Cost parameters

Since this study was conducted from the perspective of the healthcare system and adverse events were simplified, only direct medical costs were included: annual drug acquisition costs, initial hospitalization costs for cardiovascular events, and annual maintenance costs after the event. Routine follow-up costs (outpatient visits, routine laboratory tests, and basic medications) were the same for both groups and could therefore be fully offset in the incremental analysis; they were not included in the incremental analysis. Detailed cost parameters and their ranges are shown in Table 4.

**Table 4:** Detailed cost parameters.

Cost category	Value (CNY)	Range	Source
Annual cost of loxenatide	9,724.00	7,779.20–11,668.80	National Reimbursement Drug List (2023 edition)
Annual cost of sitagliptin	78.33	62.66–94.00	Yaozhi.com
Initial MI cost	41,660	33,328–49,992	(16)
Annual post-MI maintenance cost	3,476	2,781–4,171	(16)
Initial stroke cost	73,290	58,632–87,948	(16)
Annual post-stroke maintenance cost	4,512	3,610–5,414	(16)
Initial HF cost	26,883	21,506–32,260	(16)
Annual post-HF maintenance cost	6,188	4,950–7,426	(16)
Routine follow-up cost	Omitted	—	—

#### 1.3.5 Utility parameters

Health outcomes were measured in quality-adjusted life years (QALYs), calculated as the survival time in a given health state multiplied by the corresponding health utility value. Utility values were derived from previously published studies based on Chinese populations. The incremental cost-utility ratio (ICUR) was used to compare the cost per unit of utility gain between the control and experimental groups. Detailed utility parameters are presented in Table 5.

**Table 5:** Detailed utility parameters.

Health state	Utility value	Calculation	Source
S1: Event-free	0.92	—	(27)
S2: Post-MI	0.892	0.92–0.028	(16)
S3: Post-stroke	0.819	0.92–0.101	(16, 27)
S4: Post-HF	0.892	0.92–0.028	(16)
S5: Death	0	—	—

#### 1.3.6 Willingness-to-pay threshold and discounting

Following the recommendations of the China Guidelines for Pharmacoeconomic Evaluations (2020 Edition), the willingness-to-pay (WTP) threshold was set at three times the national per capita GDP in 2025 (99,700 CNY), i.e., 299,100 CNY per QALY. Both costs and health outcomes were discounted at an annual rate of 5%.

### 1.4 Sensitivity analyses

To test the robustness of the model results, one-way sensitivity analysis and probabilistic sensitivity analysis were performed.

One-way sensitivity analysis. Key parameters (drug costs, cardiovascular event costs, utility values, transition probability multipliers, hazard ratios, discount rate, time horizon, etc.) were varied individually within plausible ranges (baseline value ±20% or based on 95% confidence intervals from the literature) to observe the changes in the incremental cost-utility ratio (ICUR). Tornado plots were generated to identify the most influential parameters.

Probabilistic sensitivity analysis. Monte Carlo simulations (1,000 iterations) were conducted with simultaneous random sampling of cost parameters (Gamma distribution), probability parameters (Beta distribution), and utility parameters (Normal distribution). Incremental costs and incremental QALYs were calculated for each iteration. An incremental cost-effectiveness scatter plot and a cost-effectiveness acceptability curve (CEAC) were drawn, and the probability that loxenatide is cost-effective at the willingness-to-pay threshold was calculated (22).

Exploratory scenario analysis. To assess the potential impact of the base-case assumption that no transitions occur between non-absorbing states, we designed an exploratory scenario allowing patients with prior MI (S2) to face annual stroke and heart failure risks equal to 20% of those in the event-free population (S1), i.e., permitting S2 → S3 and S2 → S4 transitions. Comparison of this scenario with the base-case results allowed us to evaluate the direction and extent of the impact of this model simplification on the ICER.

### 1.5 Analysis software

Following the recommendations of the China Guidelines for Pharmacoeconomic Evaluations (2020 Edition), all modeling and analyses were performed using Microsoft Excel 2019 and PyCharm (2020.1.1).

## 2 Results

### 2.1 Base-case results

The base-case results are shown in Table 6. Over the 40-year simulation, the loxenatide group gained 1.9476 additional QALYs compared with the sitagliptin group, while incurring an additional total cost of 159,817.23 CNY. The resulting incremental cost-effectiveness ratio (ICER) was 82,058.77 CNY per QALY, which is far below the predefined willingness-to-pay threshold (299,100 CNY per QALY). Therefore, loxenatide is cost-effective compared with sitagliptin (Figure 1).

**Table 6:** Base-case results.

Parameter	Sitagliptin group	Loxenatide group	Increment
Cost (CNY)	43,565.27	203,382.50	159,817.23
QALY	13.2801	15.2277	1.9476
ICER (CNY/QALY)	–	–	82,058.77

### 2.2 One-way sensitivity analysis results

**Figure 2:**
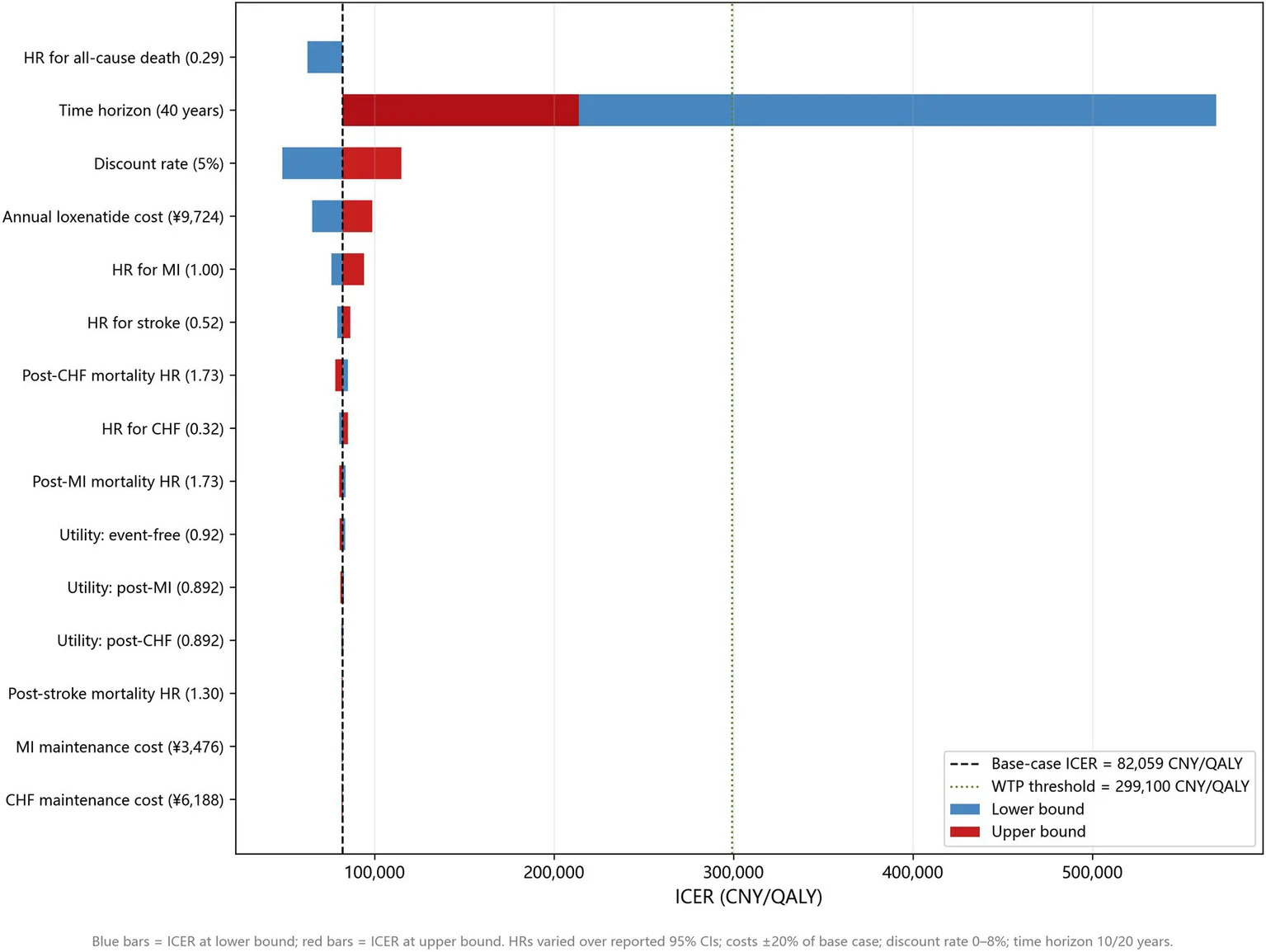
One-way sensitivity analysis results.

To examine the robustness of the model results, one-way sensitivity analysis was performed on key parameters, and a tornado plot was generated (Figure 2). Figure 2 shows that the five parameters with the greatest impact on the ICER were, in descending order: (1) the hazard ratio for all-cause death (HR_Death, 0.29), whose upper CI bound (1.27) drove the ICER toward infinity in some simulations—indicating that mortality reduction is the core driver of loxenatide’s cost-effectiveness; (2) the time horizon, which, when shortened to 10 years, increased the ICER to 568,866 CNY/QALY—reflecting the clinical reality that cardiovascular benefits of GLP-1 RAs require a medium-to-long-term horizon to fully materialize; (3) the discount rate, which, when varied from 0 to 8%, yielded ICERs ranging from 48,608 to 115,057 CNY/QALY, all below the WTP threshold; (4) the annual cost of loxenatide (9,724 CNY), which, over a ± 20% range, produced ICERs between 65,286 and 98,832 CNY/QALY; and (5) the hazard ratio for myocardial infarction (HR_MI, 1.00), whose wide 95% CI (0.49–2.41) resulted in ICERs from 76,115 to 94,249 CNY/QALY.

Parameters with smaller impacts included: HR_Stroke, post-HF mortality HR, HR_CHF, post-MI mortality HR, utility values, complication costs, post-stroke mortality HR, and sitagliptin cost. Complication costs each affected the ICER by less than 1,000 CNY/QALY, indicating that the results are insensitive to the precision of cost estimates for complications. The discount rate pattern (lower rates yielding lower ICERs) is consistent with health economics theory, as zero discounting preserves the full weight of long-term QALY gains from cardiovascular protection.

Overall, except for two extreme scenarios—a 10-year horizon (ICER = 568,866 CNY/QALY) and the upper bound of the death HR (1.27)—all parameters, when varied within plausible ranges, produced ICERs far below the WTP threshold (299,100 CNY/QALY). With a 20-year horizon, the ICER was 213,753 CNY/QALY, still below the WTP threshold. Across all 21 sensitivity parameters, the base-case conclusion remains highly robust: loxenatide consistently demonstrates a cost-effectiveness advantage over a wide range of parameter uncertainty (23).

### 2.3 Probabilistic sensitivity analysis results

**Figure 3:**
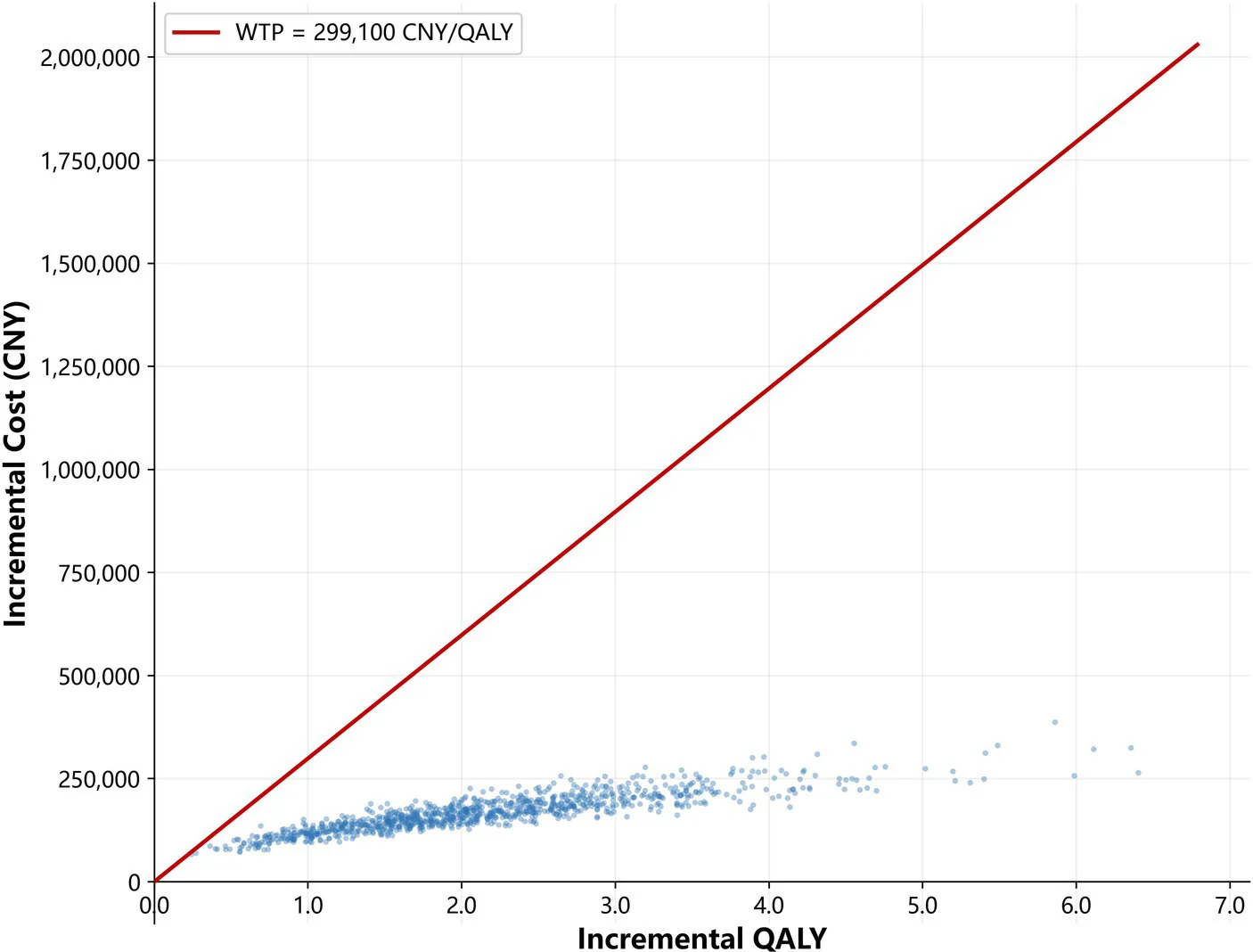
Incremental cost-utility scatter plot.

To comprehensively assess the impact of parameter uncertainty on the study conclusions, a probabilistic sensitivity analysis (PSA) was performed. Monte Carlo simulations (1,000 iterations) were used to simultaneously sample all key model parameters (drug costs, complication treatment costs, health utilities, hazard ratios, discount rate, etc.). An incremental cost-effectiveness scatter plot (Figure 2) and a cost-effectiveness acceptability curve (CEAC) (Figure 3) were generated to quantify the influence of parameter variability on the cost-effectiveness of the loxenatide strategy.

#### 2.3.1 Incremental cost-utility scatter plot

In Figure 3, the horizontal axis represents the incremental quality-adjusted life years (ΔQALY) of loxenatide versus sitagliptin, indicating the health gain, and the vertical axis represents the incremental cost (ΔC), indicating the economic burden. The red line is the willingness-to-pay (WTP) threshold (299,100 CNY/QALY); points below the line are considered cost-effective, while those above are not. Figure 3 shows:

① All 1,000 simulation points lie to the right of the vertical axis (ΔQALY>0), indicating that no simulation resulted in a reversal of efficacy. Across the entire range of parameter uncertainty, loxenatide consistently provided greater health benefits than sitagliptin. This finding is consistent with the clinical evidence from the FIGHTING-2 study, in which loxenatide significantly reduced the risks of stroke (HR = 0.52), heart failure hospitalization (HR = 0.32), and all-cause death (HR = 0.29), thereby supporting the clinical validity of the model.
② All points are located in the first quadrant (ΔC > 0, ΔQALY>0),indicating that in every simulated scenario, loxenatide was associated with additional health gains at an additional cost, as expected given its higher annual drug cost (9,724 CNY/year). No cost-saving (fourth quadrant) scenario was observed, which may be related to the high drug cost of loxenatide and the specified ranges of cost variability.
③ All points lie below the WTP line, meaning that in 100% of the simulations, the ICER was lower than the WTP threshold (ΔC/ΔQALY<299,100 CNY/QALY). Thus,loxenatide is unequivocally cost-effective compared with sitagliptin across the entire range of parameter uncertainty.

#### 2.3.2 Cost-effectiveness acceptability curve

**Figure 4:**
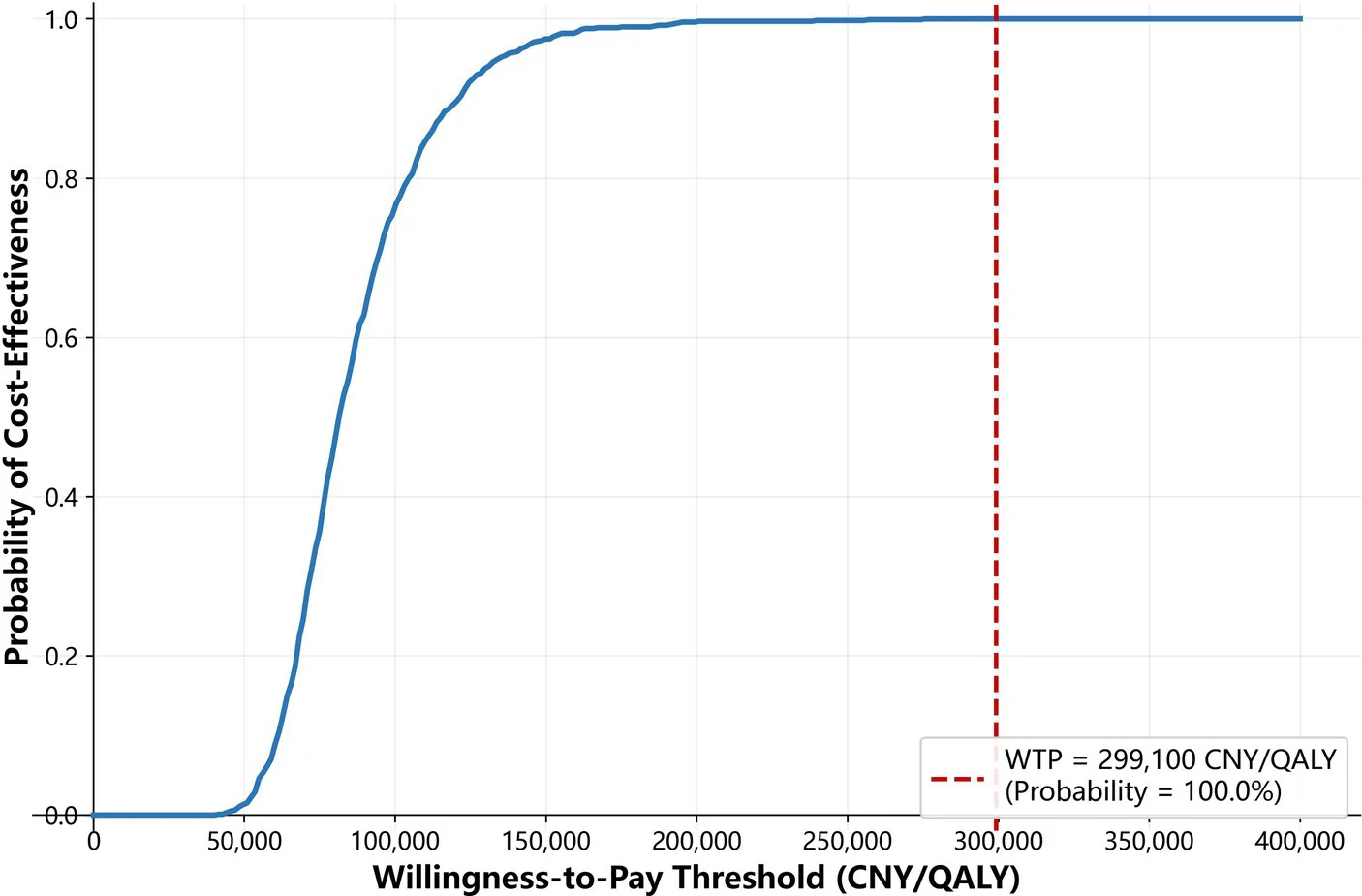
Cost-effectiveness acceptability curve (CEAC).

Figure 4 presents the probability that loxenatide is cost-effective at different willingness-to-pay (WTP) thresholds. When the WTP threshold is 0, the probability is 0; as the threshold increases, the probability rises rapidly. At a WTP threshold of 299,100 CNY/QALY, the probability that loxenatide is cost-effective reaches 100%, which is fully consistent with the observation in the scatter plot that all points lie below the WTP line. Even when the WTP threshold is lowered to 100,000 CNY/QALY, the probability remains close to 100%, indicating that the model results are insensitive to the choice of the WTP threshold and that the economic advantage of loxenatide is extremely robust.

### 2.4 Exploratory scenario analysis

In the base-case model, patients with prior myocardial infarction (S2) could only transition to death (S5), without accounting for the possibility of subsequent stroke or heart failure. In clinical practice, however, these patients are at significantly higher risk of both events due to shared atherosclerotic pathology and impaired cardiac function. To test whether the omission of this pathway affected the robustness of our conclusions, we conducted an exploratory scenario analysis.

In this scenario, patients in the S2 state were assumed to face annual risks of stroke and heart failure equal to 20% of the corresponding risks in the event-free population (S1) of the same cycle. This assumption was based on: (1) the fact that patients with prior MI receive secondary prevention therapies (antiplatelets, statins, beta-blockers, etc.), which reduce their subsequent risks compared with untreated populations; and (2) the use of a conservative 20% multiplier to test whether the conclusion remained robust even when this additional pathway was explicitly modeled.

Based on the above assumptions, the following extensions were made to the state transition equations: (1) two additional transition pathways were added from S2 to S3 and S2 to S4; (2) patients transitioning via S2 → S3 or S2 → S4 incurred the corresponding first-event hospitalization costs, followed by annual maintenance costs for the respective states; and (3) all other transition rules remained unchanged from the base-case model.

The results of the scenario analysis, alongside those of the base-case model, are presented in Table 7.

**Table 7:** Results of the scenario analysis.

Parameter	Base-case model	Scenario analysis	Change
Cost – sitagliptin group (CNY)	43,565.27	45,115.91	+1,550.64
Cost – loxenatide group (CNY)	203,382.50	204,955.81	+1,573.31
Incremental cost ΔC (CNY)	159,817.23	159,839.89	+22.67
QALY – sitagliptin group	13.2801	13.2810	+0.0009
QALY – loxenatide group	15.2277	15.2237	−0.0041
Incremental QALY ΔQ	1.9476	1.9427	−0.0049
ICER (CNY/QALY)	82,058.77	82,277.92	+219.15 (+0.3%)

As shown in Table 7, when patients with prior MI were allowed to experience subsequent stroke or heart failure at 20% of the baseline population risk, the ICER increased marginally from 82,058.77 to 82,277.92 CNY/QALY, a change of only 0.3%. The changes in both costs and QALYs were minimal across groups (incremental cost increased by only 22.67 CNY, and incremental QALY decreased by only 0.0049), far from affecting the conclusion.

This can be attributed to several factors. First, the incidence of myocardial infarction itself is low (1.2% in year 1, rising to 9.5% by year 40), so only a limited proportion of patients enter the S2 state. Second, under the 20% multiplier, the absolute annual probabilities of S2 → S3 and S2 → S4 are extremely low (for example, at year 20, the baseline probabilities of stroke and heart failure in the event-free population are 2.9 and 3.6%, respectively; after adjustment by the 0.20 multiplier, these become only 0.58 and 0.72%). Third, because the additional S2 → S3/S4 probabilities are regulated by the same treatment-effect HRs (with loxenatide conferring significant protective effects), the relative difference between the two groups remains almost unchanged.

In conclusion, even when the additional risk pathway of subsequent stroke or heart failure after myocardial infarction was considered, loxenatide remained clearly cost-effective (ICER = 82,277.92 CNY/QALY, far below the WTP threshold of 299,100 CNY/QALY). The simplifying assumption in the base-case model did not materially affect the study conclusions, demonstrating that the model results are highly robust.

### 2.5 Scenario analysis with alternative discount rates

To further test the impact of the discount rate on the base-case conclusions, we added two additional scenarios with discount rates of 0 and 3%, in addition to the 5% rate used in the base-case analysis, following international practice. The results are presented in Table 8. Across all three discount rates, loxenatide remained cost-effective relative to sitagliptin, with all ICERs falling far below the WTP threshold of 299,100 CNY/QALY.

**Table 8:** Discount rate scenario analysis results.

Discount rate	Sitagliptin cost (CNY)	Loxenatide cost (CNY)	ΔC (CNY)	Sitagliptin QALY	Loxenatide QALY	ΔQALY	ICER (CNY/QALY)
0%	96,904.46	445,740.17	348,835.71	24.1947	31.3713	7.1766	48,607.63
3%	58,335.76	268,238.20	209,902.44	16.4294	19.6177	3.1883	65,834.77
5%	43,565.27	203,382.50	159,817.23	13.2801	15.2277	1.9476	82,058.77

## 3 Discussion

Based on the findings of the FIGHTING-2 study and the inclusion criteria of the FLYING trial, this study used a five-state Markov model to evaluate, for the first time from the Chinese healthcare system perspective, the long-term cost-utility of loxenatide (0.2 mg once weekly) versus sitagliptin (100 mg once daily) in patients with type 2 diabetes at high cardiovascular risk. Over a 40-year time horizon, loxenatide provided an additional 1.9476 QALYs per patient at an incremental cost of 159,817.23 CNY, resulting in an ICER of 82,058.77 CNY per QALY. This value is well below the willingness-to-pay threshold (three times China’s per capita GDP in 2025, i.e., 299,100 CNY per QALY), indicating that loxenatide is clearly cost-effective. One-way sensitivity analysis showed that the ICER remained below the threshold when all key parameters were varied within plausible ranges; the hazard ratio for heart failure (HR_CHF) had the greatest impact on the ICER, but it did not reverse the conclusion. In probabilistic sensitivity analysis, all 1,000 Monte Carlo simulation points fell below the WTP line, and the probability of loxenatide being cost-effective was 100%, fully demonstrating the robustness of the model results.

Currently, long-term pharmacoeconomic evaluations of GLP-1 receptor agonists versus DPP-4 inhibitors in patients with type 2 diabetes at high cardiovascular risk are still limited. Existing studies have confirmed that some GLP-1 receptor agonists (e.g., semaglutide, dulaglutide) are superior to DPP-4 inhibitors in reducing cardiovascular events and are cost-effective in Chinese populations. Gu et al. based on the SUSTAIN China trial, once-weekly semaglutide (0.5 mg or 1 mg) provided additional QALY gains of 0.08 and 0.12, respectively, while reducing total costs, demonstrating absolute dominance (15). Our results are consistent with these findings but show a larger QALY gain (ΔQALY = 2.08) for loxenatide, mainly because: (1) our simulation had a longer time horizon (40 years), fully capturing the cumulative effects of long-term cardiovascular protection; (2) loxenatide demonstrated more substantial risk reductions in the FIGHTING-2 study for stroke (HR = 0.52), heart failure hospitalization (HR = 0.32), and all-cause death (HR = 0.29), whereas the corresponding HRs for semaglutide were higher (e.g., stroke HR = 0.63). Furthermore, a short-term cost analysis in a Spanish population showed that GLP-1 receptor agonists had a cost advantage over DPP-4 inhibitors for the composite endpoint (HbA1c < 7% with no weight gain and no hypoglycemia) (24). Our study provides the first long-term economic evidence based on Chinese real-world data (FIGHTING-2), filling a gap in this area for loxenatide.

The substantial QALY gain with loxenatide is primarily driven by its great improvement in cardiovascular outcomes. The FIGHTING-2 study showed that loxenatide reduced the risk of 3P-MACE (non-fatal myocardial infarction, non-fatal stroke, and cardiovascular death) by 32% compared with the control group, with reductions of 37% for non-fatal stroke, no significant difference for non-fatal myocardial infarction, and 44% for cardiovascular death. These benefits directly translate into fewer major adverse events (myocardial infarction, stroke, heart failure hospitalization), thereby avoiding high acute-phase treatment costs and subsequent annual maintenance costs. Although the annual drug cost of loxenatide (9,724 CNY) is much higher than that of sitagliptin (78.33 CNY), the substantial reduction in complication rates prevented a large increase in total long-term costs (incremental cost over 40 years only 159,817.23 CNY). Moreover, loxenatide showed additional benefits in weight loss (mean reduction 2.16 kg) and blood pressure improvement (systolic blood pressure reduction 6.6 mmHg) in FIGHTING-2. Although these factors were ignored in the base-case analysis due to the lack of Chinese utility weights for BMI changes, they further strengthen the clinical value of loxenatide. Notably, omitting BMI-related utility changes is a conservative approach because weight loss generally improves quality of life; if included, the QALY gain for loxenatide might be even higher.

This study has several strengths. First, the model was based on the UKPDS 82 risk equations, a gold standard for predicting long-term complications in type 2 diabetes, and was calibrated with Chinese population data (baseline characteristics from the SUSTAIN China trial), ensuring the validity of transition probability extrapolation. Second, clinical efficacy parameters were directly derived from the FIGHTING-2 real-world study, which enrolled 12,341 Chinese patients and provided HRs for stroke, heart failure, and death, thereby avoiding indirect-comparison bias. Third, cost and utility data were all sourced from Chinese local sources (National Reimbursement Drug List, centralized procurement prices, Chinese EQ-5D-3L decrement studies, etc.),closely reflecting the actual health economics context in China. Fourth, comprehensive one-way and probabilistic sensitivity analyses confirmed the robustness of the conclusions; in particular, PSA showed that loxenatide was cost-effective in 100% of simulations, indicating insensitivity to parameter uncertainty.

Nevertheless, this study has several limitations. First, the FIGHTING-2 study has been published only as an abstract and does not provide detailed baseline characteristics (e.g., distributions of age, HbA1c, and diabetes duration). Therefore, the inclusion criteria for our target population were primarily based on the FLYING and SUSTAIN China trials, which may introduce some population heterogeneity. Second, the UKPDS 82 equations were developed using a UK population; although widely used globally, their predictive accuracy in the Chinese population needs to be validated in real-world studies. Third, for patients who have experienced myocardial infarction, stroke, or heart failure, the subsequent annual mortality probability was estimated using hazard ratios rather than directly by the UKPDS equations. The precision of these HRs depends on the adjustment factors of the original studies and may not perfectly match our study population. Fourth, the model did not incorporate dynamic feedback effects of changes in blood glucose, blood pressure, and body weight on the risk equations; instead, we used HRs to directly adjust annual event probabilities. This simplified mechanistic pathway may underestimate the long-term benefits of loxenatide mediated by sustained improvements in metabolic parameters. Fifth, only direct medical costs (drugs, complications, hospitalizations, and maintenance costs) were included; indirect costs (e.g., productivity loss, caregiver costs) and intangible costs (e.g., pain, psychological burden) were not considered, nor were routine diabetes management costs (omitted because they were the same in both groups). This may lead to some underestimation of total costs, but since the cost difference between groups mainly comes from drugs and complications, the impact on the ICER is limited. Sixth, we only compared loxenatide with sitagliptin, not with other DPP-4 inhibitors (e.g., linagliptin, alogliptin) or other GLP-1 receptor agonists (e.g., semaglutide, dulaglutide, liraglutide). Thus, our conclusions cannot be directly extrapolated to broader classes of glucose-lowering drugs. The model did not include microvascular complications (e.g., nephropathy, retinopathy). Given the study’s focus on a high cardiovascular risk population, the impact on the incremental ICER is expected to be limited; if loxenatide confers cardio-renal benefits, the current estimates are likely conservative.

Future research could be improved in several ways: (1) further calibrate model parameters based on the full publication of the FIGHTING-2 study, especially obtaining more precise subgroup HRs; (2) conduct long-term follow-up studies in Chinese real-world populations to validate the calibration coefficients of the UKPDS equations for Chinese patients; (3) include more cost dimensions (e.g., indirect costs) and utility measures (e.g., BMI-related health utilities) to build a more comprehensive societal perspective model; and (4) expand the comparator drugs to include cardiorenal protective agents (e.g., SGLT-2 inhibitors) in combination or sequential strategies, providing richer evidence for clinical decision-making.

## 4 Conclusion

In conclusion, from the perspective of the Chinese healthcare system, loxenatide is cost-effective compared with sitagliptin for the treatment of patients with type 2 diabetes at high cardiovascular risk over a 40-year simulation period, and sensitivity analyses confirm the robustness of this conclusion. Therefore, loxenatide can be considered a high-value option for optimizing glycemic and cardiovascular risk management in this patient population. It is recommended that reimbursement policies be adjusted to facilitate its use, thereby improving the allocation of limited healthcare resources.

## Data Availability

The original contributions presented in the study are included in the article/supplementary material, further inquiries can be directed to the corresponding author/s.
